# *CDKN2A/2B* gene variants and gene-environment interactions increase the risk of gestational diabetes mellitus in Chinese women

**DOI:** 10.3389/fcell.2026.1725802

**Published:** 2026-04-22

**Authors:** Yi Zhang, Runqiu Yang, Xin Wang, Hongsong Yu

**Affiliations:** 1 Department of Immunology, Special Key Laboratory of Gene Detection and Therapy of Guizhou Province, Zunyi Medical University, Zunyi, China; 2 The People’s Hospital of QianNan, Duyun, China

**Keywords:** CDKN2A/2B, diabetes, gene, gestational, interaction, mellitus, O3, PM2.5

## Abstract

**Introduction:**

This study investigates the association between the cyclin-dependent kinase inhibitor 2A/2B (*CDKN2A/2B*) gene and gestational diabetes mellitus (GDM) in Chinese women, with a focus on potential gene-environment interactions.

**Methods:**

A case-control study was conducted with 1,566 pregnant women from Beijing, Wuhan, and Zunyi, China. Blood samples were analyzed using targeted next-generation sequencing. Logistic regression was applied to examine the relationship between gene polymorphisms, environmental exposures, and GDM. Gene-environment interactions were assessed using Excel tools developed by Knol and Andersson et al.

**Results:**

Genetic analysis revealed significant associations between two of the three loci: rs23832080 (*P* = 0.002, OR = 0.801) and rs10811661 (P = 0.00055, OR = 0.746), and GDM. Haplotype analysis showed that haplotypes of these loci were correlated with GDM (AGC: P = 0.002, OR = 0.784; AAT: *P* = 0.024, OR = 1.183; GAT: *P* = 0.043, OR = 1.247). Environmental exposure analysis indicated that ozone (O_3_) and PM_2.5_ were associated with GDM. Interaction analysis revealed that the wild-type alleles of rs2383208 and rs10811661 interacted with O_3_, increasing the risk of GDM, while the wild-type allele of rs10811661 also interacted with PM_2.5_, further elevating GDM risk.

**Conclusion:**

CDKN2A/2B gene polymorphisms, haplotypes, environmental exposure to O_3_ and PM_2.5_, and gene-environment interactions contribute to an increased risk of GDM in Chinese women.

**Trial Registration:**

Chinese Clinical Trial Registry, chictr.org.cn, ChiCTR2000029178.

## Graphical Abstract

**Figure:**
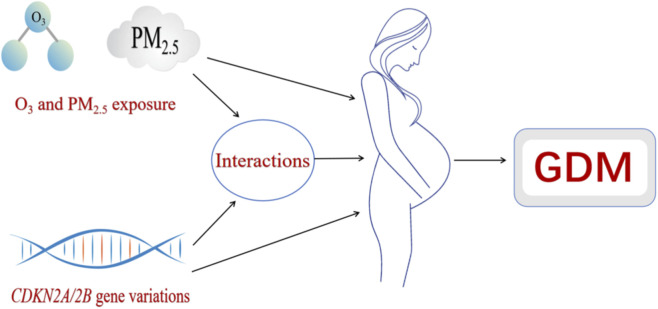
Synopsis: Research on the interaction between genetics and environmental factors is still limited. This study highlights the complexity of gene-environment interactions and emphasizes the need to consider gene regulation when assessing the impact of environmental factors on disease risk.

## 1 Introduction

Gestational diabetes mellitus (GDM), a prevalent and high - risk complication during pregnancy, can have negative impacts on the health of both the mother and the fetus. GDM is defined as a condition that either manifests during pregnancy or is first diagnosed at this time. In recent years, in China, the occurrence rate of GDM has been increasing steadily. Women with GDM are at a higher risk of severe perinatal complications, such as excessive amniotic fluid, gestational hypertension, and preterm delivery. Their infants are also more vulnerable to conditions like neonatal respiratory distress syndrome and macrosomia (Johns et al., 2018). Compared to women with normal pregnancies, those with GDM are at an elevated risk of developing type 2 diabetes after delivery (Moon et al., 2017). This highlights the significant health risks GDM poses to both mothers and their children. Despite the general belief that GDM is associated with insulin resistance and hyperinsulinemia, the exact mechanisms governing its development remain poorly understood (Liu et al., 2022). Nevertheless, newly emerging evidence indicates that genetic factors are of crucial importance in the occurrence of GDM (Wei et al., 2021; Yang et al., 2025b).

The CDKN2A/2B gene, located on the short arm of chromosome 9, includes two protein-coding genes within the 9p21.3 locus. Variations in this gene have been associated with several types of cancer, coronary artery disease, and other cardiovascular diseases (Wei et al., 2022; Jensen et al., 2022). Moreover, research has found links between genetic variations in CDKN2A/2B and diabetes. For instance, Kong et al. (2018) identified that polymorphisms in the CDKN2A/2B region might influence the risk of type 2 diabetes by affecting gene expression and β-cell proliferation. Similarly, Amit et al. (Verma et al., 2021) found in case-control studies in India that genetic variants in multiple genes, including CDKN2A/2B, were associated with the onset of type 2 diabetes. Given the importance of CDKN2A/2B in GDM, further research is needed in the Chinese population to fill the current research gap in this area.

In addition to genetic factors, environmental influences also contribute to the development of GDM. Recent studies have highlighted the impact of air pollution on human health, especially due to environmental degradation. PM2.5, a major air pollutant, poses a significant health risk and is strongly associated with respiratory, cardiovascular, and cerebrovascular diseases. Research has shown that PM2.5 is not only linked to higher fasting blood sugar levels but also has a causal relationship with GDM. As PM2.5 concentrations rise, so does the risk of developing GDM (Shin et al., 2020; Li et al., 2019; Yang et al., 2023). Ozone (O3), another air pollutant, has also been shown to impact human health, particularly by influencing blood glucose levels. Early studies have found a correlation between increased O3 exposure and higher fasting blood glucose and glycated hemoglobin levels (Chuang et al., 2010). Some experiments suggest that O3 exposure could damage beta cells, leading to insulin resistance (Miller et al., 2016). A cohort study in Shanghai, China, found that O3 exposure during pregnancy disrupts glucose homeostasis and increase the risk of GDM, with the critical window for its impact occurring between the 5th and 10th weeks of pregnancy (Zhang et al., 2023a).

GDM is a complex disease shaped by various factors, including genetic and environmental interactions. Thus, this study aims to explore the relationship between CDKN2A/2B gene polymorphisms and the risk of GDM in Chinese pregnant women, while also investigating the specific effects of gene-environment interactions on GDM development.

## 2 Materials and methods

### 2.1 Study participants

Participants in this study were selected according to the diagnostic criteria set by the International Association for Diabetes and Pregnancy (IADPSG). During the 24th to 28th weeks of pregnancy, a glucose tolerance test was carried out. In this test, fasting blood glucose (FPG) was measured, along with the glucose levels 1 h (1 h - PG) and 2 hours (2 h - PG) following a 75 - gram oral glucose challenge. GDM was diagnosed if any of the following thresholds were met: FPG ≥ 5.1 mmol/L, 1 h-PG ≥ 10.0 mmol/L, or 2 h-PG ≥ 8.5 mmol/L. Participants with pregestational diabetes, multiple pregnancies, or other endocrine disorders affecting blood glucose regulation were excluded. The final sample comprised 1,566 participants, including 703 GDM patients and 863 healthy controls. This study was approved by the Ethics Committee of Zunyi Medical University, and all participants provided informed consent. Participants were excluded if they had: (1) preexisting diabetes mellitus; (2) pregnancy-related complications, such as gestational hypertension or thyroid dysfunction; (3) significant diseases of major organs, including severe cardiovascular, hepatic, renal, or psychiatric disorders; or (4) diagnosed immune system disorders.

### 2.2 Selection of single nucleotide polymorphisms (SNPs)

After reviewing relevant domestic and international literature and considering the significant role of CDKN2A/2B mutations in diabetes mellitus (Kasuga et al., 2017; Binh et al., 2015; Tarnowski et al., 2017a; Wang et al., 2011; Wang et al., 2015; Hertel et al., 2008) (Supplementary Table S1), three SNPs within the CDKN2A/2B gene, including rs1063192, rs2383208, and rs10811661, were selected for this study. We also calculated pairwise linkage disequilibrium statistics (r^2^ and D') for three SNPs: rs10811661, rs2383208, and rs10757283.

### 2.3 DNA extraction and sequencing

In this study, genomic DNA was isolated from all samples by making use of the Magnetic Universal Genomic DNA Kit (DP705, Tiangen Biochemical Technology Co., Ltd., Beijing, China). To guarantee the quality of DNA extraction, library construction, and sequencing outcomes, the sequencing depth was set at 100X and the clean Q30-all >80%. iGeneTech Bioscience Co., Ltd. (Beijing, China) carried out the sequencing work, applying second - generation targeted sequencing technology. This technology utilizes specific probes to capture the region of interest in the genomic DNA and then sequence it, which allows for the accurate identification of SNPs in each sample. Subsequently, the obtained data were aligned with a reference genome (GRCh37/hg19) for the purpose of variant identification and genotype determination. The Illumina high - throughput sequencing platform was utilized during this process.

### 2.4 Extraction of environmental pollutant data

Environmental exposure data, including daily PM_2.5_ concentrations and maximum 8-h average O_3_ concentrations, were obtained from the TAP website (http://tapdata.org.cn/) based on the participants' residential addresses. The data were provided at a 10 km × 10 km resolutio.

### 2.5 Statistical analysis

Statistical analyses were conducted using SPSS 29.0 software (SPSS, Inc., Chicago, IL, United States). Differences between the case and control groups were assessed using either the T-test or the rank sum test, depending on data distribution. Allele frequencies were analyzed using the Chi-square test. Logistic regression was used to construct and analyze dominant, recessive, and additive models, adjusting for covariates such as BMI, age, and pregnancy status. A significance level of 0.05 was set for determining statistical significance. Hardy-Weinberg equilibrium (HWE) was assessed using the Chi-square test, and any control group with a P-value below 0.05 was excluded from further analysis. Haplotype analysis was performed using the online “SHEsis” tool. In addition, we further characterized gene–environment interaction on the additive scale using the relative excess risk due to interaction (RERI), the attributable proportion due to interaction (AP), and the synergy index (S). We calculated these measures and their 95% confidence intervals (CIs) with an Excel-based calculator that implements the delta method for CI estimation (Knol and VanderWeele, 2012; Andersson et al., 2005). In this analysis, genetic variation and environmental factors were dichotomized (e.g., carriers of risk alleles versus non-carriers; high BMI versus low BMI based on clinically relevant cutoffs) to create four exposure groups. The groups were coded as follows: (1) not exposed to both factors (0, 0), (2) exposed to environmental factors only (0, 1), (3) exposed to genetic factors only (1, 0), and (4) exposed to both factors (1, 1). If the RERI or AP was greater than 0, or S was greater than 1, and the 95% CIs for these estimates did not include zero (RERI and AP were 0, and S was 1), then additive interaction was considered to exist. The principles underlying additive interaction are summarized in Supplementary Table S2.

## 3 Results

### 3.1 Demographics and clinical characteristics

Supplementary Table S3 provides a summary of the demographic and clinical characteristics of the study participants. These participants are the same as those in our previous study (Huang et al., 2024; Yang et al., 2025b). No significant difference in height was observed between the case and control groups (*P* > 0.05). Conversely, significant differences emerged in terms of pre - pregnancy body mass index (BMI), age, gestational age, fasting blood glucose, as well as 1 - hour and 2 - hour postprandial blood glucose levels (*P* < 0.05). The case group exhibited higher BMI, age, and gestational age, all of which are established risk factors for elevated blood glucose levels. These factors were subsequently included as covariates in the association analysis. Our previous research has summarized the demographic and clinical characteristics of the study participants.

### 3.2 Association analysis of SNPs and haplotypes with GDM

Data for all analyzed loci are provided in Supplementary Table S4. HWE calculations showed no deviation in the control groups (*P* > 0.05), confirming that they met equilibrium criteria. To investigate the linkage disequilibrium (LD) and genetic effects at the three loci, we first calculated the pairwise LD among them. The analysis revealed that rs1063192 and rs2383208 exhibited low LD (r^2^ = 0.004, D' = 0.51, Supplementary Table S5), as did rs1063192 and rs10811661 (r^2^ = 0.003, D' = 0.44, Supplementary Table S5), indicating that these pairs are in linkage equilibrium and represent completely independent genetic markers. In contrast, rs2383208 and rs10811661 showed high LD (r^2^ = 0.79, D' = 0.93, Supplementary Table S5), suggesting they belong to the same haplotype block and are in moderate linkage disequilibrium. Model analysis revealed that two loci in the *CDKN2A/2B* gene were significantly associated with GDM (*P* < 0.05). After adjusting for pre-pregnancy BMI, age, and number of pregnancies, both rs2383208 and rs10811661 remained significantly associated with GDM (*P* < 0.05), as detailed in Table 1. Further analysis indicated that the wild-type and A allele of rs2383208, as well as the wild-type and T allele of rs10811661, were associated with an increased risk of GDM (*P* < 0.05, OR > 1). Conversely, the mutant genotypes of rs2383208 and rs10811661, along with the G and C alleles, were linked to a reduced risk of GDM (*P* < 0.05, 0 < OR < 1), as shown in Supplementary Tables S6, S7. Haplotype analysis of these loci revealed that the AGC haplotype at rs1063192-rs2383208-rs10811661 was associated with a decreased risk of GDM (*P* = 0.002, OR = 0.784, 95% CI: 0.673–0.913), while the AAT and GAT haplotypes were linked to an increased risk of GDM (*P* = 0.024, OR = 1.183, 95% CI: 1.023–1.368; *P* = 0.043, OR = 1.247, 95% CI: 1.006–1.545), as shown in Table 2. And the global P-value for the haplotype block is 0.003.

**TABLE 1:** Association between candidate SNPs and GDM.

Gene	SNP	Model	*P-*value	OR (95% CI)	* *P-*value	OR (95% CI)*
CDKN2A/2b	**rs1063192**	Dominant	0.25	0.763 (0.481–1.210)	0.239	0.755 (0.474–1.204)
		Recessive	0.967	1.004 (0.816–1.236)	0.834	1.023 (0.828–1.264)
		Additive	0.235	0.755 (0.475–1.200)	0.248	0.758 (0.474–1.213)
CDKN2A/2b	**rs2383208**	Dominant	**0.03**	**0.794(0.645–0.978)**	**0.014**	**0.767(0.621–0.947)**
		Recessive	**0.006**	**0.691(0.531–0.899)**	**0.012**	**0.707(0.541–0.926)**
		Additive	**0.002**	**0.637(0.476–0.852)**	**0.003**	**0.635(0.472–0.856)**
CDKN2A/2b	**rs10811661**	Dominant	**0.002**	**0.718(0.581–0.887)**	**0.001**	**0.698(0.563–0.866)**
		Recessive	**0.001**	**0.642(0.498–0.828)**	**0.001**	**0.649(0.501–0.840)**
		Additive	**0.00008**	**0.563(0.423–0.749)**	**0.000085**	**0.558(0.417–0.746)**

* P-value, corrected for BMI, age, pregnancy and OR. Bold values indicate statistical significance (P < 0.05).

**TABLE 2:** Association of specific haplotypes with GDM.

Gene	SNPs included	Haplotype	Fisher’s p	OR	95% CI
CDKN2A/2B	rs1063192|rs2383208 rs10811661	**A A T**	**0.024**	**1.183**	**1.023–1.368**
	rs1063192|rs2383208 rs10811661	**A G C**	**0.002**	**0.784**	**0.673–0.913**
	rs1063192|rs2383208 rs10811661	**G A T**	**0.043**	**1.247**	**1.006–1.545**
	rs1063192|rs2383208 rs10811661	G G C	0.220	0.835	0.625–1.115

All those frequnecy <0.03 willl be ignored in analysis. Bold values indicate statistical significance (P < 0.05).

### 3.3 Analysis of the environmental exposure factors

The entire gestational period is critical for fetal growth and development, with the fetus being particularly vulnerable to environmental pollutants due to the incomplete development of organs and systems (Selevan et al., 2000). Early pregnancy, in particular, is a key period for the initial development of various organs and the nervous system, and exposure to harmful environmental factors during this time can significantly impact later development. Research suggests that exposure to certain environmental pollutants in the first trimester may increase the risk of GDM (Yu et al., 2023). This study examined environmental exposure throughout pregnancy, focusing specifically on early pregnancy (1–13 weeks). As shown in Table 3, analysis across the entire gestational period revealed that only O_3_ exposure was significantly associated with GDM, with an increase in GDM incidence as O_3_ concentrations rose (*P* < 0.05, OR > 1). During early pregnancy, both O_3_ and PM_2.5_ were found to be associated with GDM. Higher O_3_ concentrations were linked to a reduced risk of GDM (*P* < 0.05, OR < 1), while higher PM_2.5_ concentrations were associated with an increased risk of GDM (*P* < 0.05, OR > 1). Given the complex composition of PM_2.5_, a further analysis was conducted to examine the correlation between the specific components of PM_2.5_ and GDM, with results presented in Supplementary Table S8. Notably, exposure to O_3_ during the early pregnancy was associated with a reduced risk of GDM. We hypothesize that this observed protective effect may be attributed to the anti-inflammatory properties of O_3_. Alternatively, this phenomenon could be explained by a compensatory defense mechanism of the body. In contrast, O_3_ exposure during the whole preconception was associated with an increased risk of GDM, potentially as the body implements compensatory protective measures to prevent damage to the developing fetus.

**TABLE 3:** Associations of environmental exposure factors with GDM in different periods.

Environmental factor	*P-*value	OR (95% CI)	* *P-*value	OR (95% CI)*
Whole gestational cycle				
O_3_	**1.9134E-9**	**1.035(1.023–1.047)**	**1.0162E-7**	**1.032(1.020–1.043)**
PM_2.5_	0.228	1.008 (0.995–1.020)	0.289	1.007 (0.994–1.020)
Early pregnancy				
O_3_	**5.738E-7**	**0.991(0.987–0.994)**	**3.581E-7**	**0.990(0.987–0.994)**
PM_2.5_	**3.387E-8**	**1.015(1.010–1.020)**	**3.0089E-7**	**1.014(1.009–1.019)**

* *P* values corrected for BMI, age, pregnancy and OR. Bold values indicate statistical significance (P < 0.05).

### 3.4 Interactive analysis

Several key indicators of additive interaction were assessed, including the attributable proportion due to interaction (AP), the relative excess risk due to interaction (RERI), and the synergy index (S). A significant interaction was defined as a 95% CI for RERI and AP that did not include 0, and a 95% CI for S that did not include 1. An RERI or AP value greater than 0 indicates a synergistic interaction (Nie et al., 2016; Correia and Williams, 2018).

**FIGURE 1:**
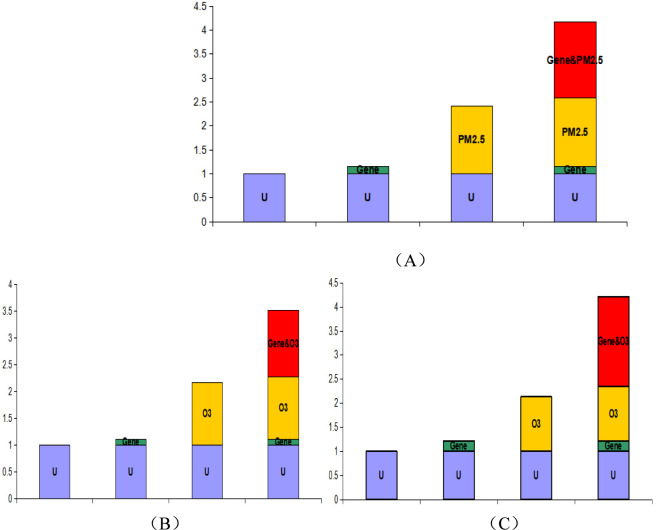
Interaction between genetic loci and environmental factors across the entire gestational cycle. This figure illustrates how specific genetic loci interact with environmental factors. **(A)** Shows the interaction between the rs10811661 TT genotype and PM_2.5_, **(B)** depicts the interaction between the rs2383208 AA genotype and O_3_, and **(C)** represents the interaction between the rs10811661 TT genotype and O_3_. In the diagram, green represents the influence of genetic factors on disease onset, yellow indicates the effect of environmental factors, and red highlights the combined impact of these interactions on disease development. The reference category is labeled as U.

**FIGURE 2:**
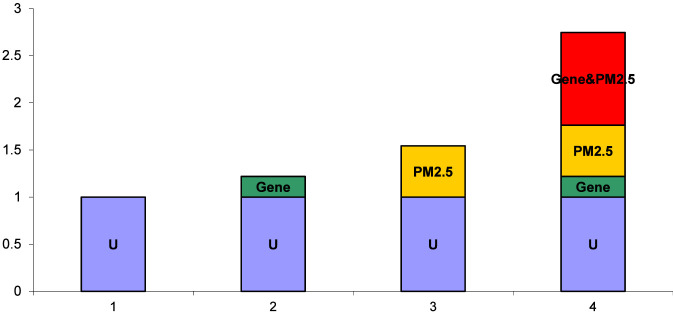
Interaction between genetic loci and environmental factors during early pregnancy. This figure illustrates the interaction between the rs10811661 TT genotype and PM_2.5._

In this analysis, two loci and environmental exposure factors associated with GDM were selected, and interaction analysis was performed using Excel tools developed by Knol and Andersson. Following the principles of additive interaction (see Supplementary Table S2), all factors were converted into binary variables, and the genetic-environmental interactions between the two loci with O_3_ and PM_2.5_ were individually analyzed. Throughout the entire gestational period, the results indicated synergistic interactions between the rs10811661 TT genotype and PM_2.5_, the rs2383208 AA genotype and O_3_, and the rs10811661 TT genotype and O_3_, all of which increased the risk of GDM (S > 1, RERI > 0). Specifically, the interaction between the rs10811661 TT genotype and PM_2.5_ increased the risk of GDM by 1.586 times (RERI = 1.586, 95% CI: 0.3866–2.7854), with the interaction effect accounting for 38.07% of the total effect (AP = 0.3807, 95% CI: 0.1704–0.5909). When both factors coexisted, the disease risk was 2.0037 times the sum of their individual effects (S = 2.0037, 95% CI: 1.1989–3.3487). Similarly, the interaction between the rs2383208 AA genotype and O_3_ increased the risk of GDM by 1.241 times (RERI = 1.241, 95% CI: 0.1981–2.2840), with the interaction effect accounting for 35.34% of the total effect (AP = 0.3534, 95% CI: 0.1303–0.5766). When both factors coexisted, the disease risk was 1.9769 times the sum of their individual effects (S = 1.9769, 95% CI: 1.1203–3.4885). Lastly, the interaction between the rs10811661 TT genotype and O_3_ increased the risk of GDM by 1.8587 times (RERI = 1.8587, 95% CI: 0.5913–3.1262), with the interaction effect accounting for 44.17% of the total effect (AP = 0.4417, 95% CI: 0.2442–0.6392). When both factors coexisted, the disease risk was 2.3775 times the sum of their individual effects (S = 2.3775, 95% CI: 1.3620–4.1504), as detailed in Table 4. Interaction graphs generated using the corresponding data in the Excel tool compiled by Andersson are presented in Figure 1. In early pregnancy, the risk of GDM due to the interaction between the rs10811661 TT genotype and PM_2.5_ was 0.9832 (RERI = 0.9832, 95% CI: 0.0816–1.8849), with the interaction effect accounting for 35.81% of the total effect (AP = 0.3581, 95% CI: 0.1090–0.6072). When both factors were present simultaneously, the disease risk was 2.2897 times the sum of their individual effects (S = 2.2897, 95% CI: 1.0343–5.0688). The interaction in early pregnancy is shown in Table 4 and Figure 2.

**TABLE 4:** Interaction values at different periods, based on the Excel tables compiled by Knol.

Interaction factor	Indicator	OR	95% CI	*P-*value
Whole gestational cycle				
rs10811661 TT and PM_2.5_				
​	RERI	1.586	0.3866–2.7854	**0.010**
​	AP	0.3807	0.1704–0.5909	**<0.001**
​	S	2.0037	1.1989–3.3487	**0.008**
rs2383208AA and O_3_				
​	RERI	1.2410	0.1981–2.2840	**0.020**
​	AP	0.3534	0.1303–0.5766	**0.002**
​	S	1.9769	1.1203–3.4885	**0.019**
rs10811661 TT and O_3_				
​	RERI	1.8587	0.5913–3.1262	**0.004**
​	AP	0.4417	0.2442–0.6392	**<0.001**
​	S	2.3775	1.3620–4.1504	**0.002**
Early pregnancy rs10811661 TT and PM_2.5_				
​	RERI	0.9832	0.0816–1.8849	**0.033**
​	AP	0.3581	0.1090–0.6072	**0.005**
​	S	2.2897	1.0343–5.0688	**0.041**

Bold values indicate statistical significance (P < 0.05).

## 4 Discussion

GDM is a common pregnancy-related disorder characterized by abnormal insulin secretion and impaired glucose metabolism (Johns et al., 2018; Alejandro et al., 2020). During pregnancy, GDM has the potential to trigger a variety of complications. It not only heightens the risk of fetal mortality but also contributes to unfavorable pregnancy outcomes. In the context of the implementation of China’s two - child and three - child policies, the incidence and influence of GDM are steadily increasing. The pathogenesis of GDM is multifactorial, influenced by genetic factors, environmental exposures, and their interactions. Identifying these factors can facilitate early detection and diagnosis of GDM in pregnant women, thus improving treatment outcomes. This study underscores the complexity inherent in the interactions between genes and the environment. It also places significant emphasis on the necessity of taking gene regulation into account while evaluating the influence that environmental factors exert on the risk of diseases.

SNPs in the 9p21.3 region of chromosome 9 have been linked to various diseases, including cancer, cardiovascular disease, insulin sensitivity, and diabetes (Spiliopoulou et al., 2022; Mahdavi et al., 2022). This region contains a long non-coding RNA and two protein-coding genes, CDKN2A and CDKN2B (Wei et al., 2022). The CDKN2A/2B genes are significant genetic factors associated with diabetes risk (Wang et al., 2015). The proteins encoded by these genes, p16INK4a and p15INK4b, inhibit the activity of cyclin-dependent kinases 4/6 (CDK4/CDK6), thereby affecting β-cell proliferation and insulin secretion (Krishnamurthy et al., 2006). Genetic variation at the CDKN2A/2B locus may be associated with the risk of type 2 diabetes and GDM by perturbing post-transcriptional and enhancer-mediated regulation (Sultana et al., 2025). The rs1063192 variant, located in the CDKN2B 3′untranslated region (UTR), could disrupt hsa-miR-323b-5p recognition and binding to the mRNA (Wang et al., 2015). rs2383208 lies in an intergenic region within the locus and may modulate expression of the long non-coding RNA ANRIL (Kong et al., 2018). rs10811661 resides within a functional enhancer and may alter its sequence and conformation, thereby affecting downstream gene expression (Kong et al., 2018). Collectively, these variants may dysregulate CDKN2A/2B expression, augment inhibition of CDK4/6, and impair pancreatic β-cell proliferation and function, rendering compensatory insulin secretion insufficient to offset gestational insulin resistance and ultimately increasing GDM risk. A nested case-control study in Japan identified a significant association between the rs2383208 locus in the CDKN2A/2B gene and the development of diabetes (Goto et al., 2018). In Poland, research on pregnant women showed that the C allele of the rs10811661 polymorphism in CDKN2A/2B was associated with a reduced risk of developing GDM, with an increasing number of C alleles acting as a protective factor (Tarnowski et al., 2017a). Wang et al. (2023) also found an association between the rs1063192 locus and GDM risk. Our study corroborates these findings and expands upon them. Our research involved a diverse population, not limited to a specific region, and incorporated both genetic model and haplotype analyses to investigate genetic factors. Additionally, environmental factors such as O3 and PM2.5 were included to assess their correlation with GDM. Employing an additive interaction model, we delved into the effects of gene - environment interactions on GDM, yielding meaningful results. This study focuses on the relationship between three SNPs (rs1063192, rs2383208, rs10811661) in CDKN2A/2B and gene-environment interactions in GDM among Chinese women. While no statistical significance was found for the rs1063192 locus, the other two sites, rs2383208 and rs10811661, showed strong associations with GDM. Consistent with other studies, the A allele of rs2383208 (P = 0.002, OR = 1.248) and the T allele of rs10811661 (P = 0.000055, OR = 1.341) increase the risk of GDM. In the haplotype analysis, several combinations of rs1063192, rs2383208, and rs10811661 were found to be associated with GDM. The “A G C” haplotype acted as a protective factor (P = 0.002, OR = 0.784), while the “A A T” and “G A T” haplotypes increased the risk of GDM (P = 0.024, OR = 1.183; P = 0.043, OR = 1.247). Our gene-environment interaction analysis revealed that throughout pregnancy, the rs10811661 TT genotype interacted with PM2.5 and O3, and the rs2383208 AA genotype interacted with O3. During early pregnancy, the rs10811661 TT genotype interacted with PM2.5, with these interactions increasing the risk of GDM (S values >1, RERI values >0). Preliminary findings also indicated a gene-environment interaction involving BMI across the entire pregnancy period and interactions between GDM and environmental factors such as O3 and PM2.5 at different stages of pregnancy. Furthermore, these results suggest that certain interactions pose a higher risk for GDM than individual genetic or environmental factors (Huang et al., 2024; Zhang et al., 2023b).

The rs10811661 site is located 125 kb upstream of CDKN2A/2B. Variation at the CDKN2A/2B locus has been associated with blood pressure, lipid profiles, and type 2 diabetes risk, potentially via effects on β-cell function. Additionally, the rs10811661 T allele has been associated with greater susceptibility to carbohydrate intolerance, which has been linked to GDM in pregnant women (Hribal et al., 2011; Tarnowski et al., 2017b). In our study, all three models analyzing rs10811661 showed an association with GDM (P < 0.05), and further research confirmed that the rs10811661 T allele increases the risk of GDM (P < 0.05, OR > 1), consistent with other findings (Kasuga et al., 2017; Binh et al., 2015). Genetic variation at rs2383208 has been implicated in impaired pancreatic β-cell function and may affect insulin secretion, with observed associations with GDM (Wang et al., 2011; Cho et al., 2009). Both rs2383208 and rs10811661 are located in the same linkage block, and their combined effects further increase the risk of GDM (Kong et al., 2018). The rs1063192 polymorphism, located within the binding site of hsa-miR-323b-5p in the CDKN2B 3′UTR, is associated with GDM. Studies suggest that miR-323b-5p may inhibit the translation of CDKN2B, and certain alleles of rs1063192 may alter this inhibition, affecting p15INK4b protein expression and ultimately influencing β-cell proliferation and diabetes risk (Wang et al., 2015; Horswell et al., 2013). Despite other studies finding an association between rs1063192 and GDM (Wang et al., 2015; Wang et al., 2023), our study did not observe similar results. No significant correlation between rs1063192 and GDM was observed, potentially due to factors such as sample size and demographic differences within the study population.

The onset of GDM is influenced by multiple factors. In addition to genetic predispositions, adverse environmental conditions also contribute to the risk. Air pollution, particularly the presence of pollutants like O3 and PM2.5, has garnered increasing attention in recent years as a significant environmental risk factor for GDM (Nazarpour et al., 2023; Susa et al., 2025). Our study found that both O3 and PM2.5 are significantly associated with GDM, with higher concentrations correlating with an increased risk (P < 0.05, OR >1). These findings are consistent with those of Zhang et al. (2023a) and Nazarpour et al. (2023). However, some studies have reported opposite results regarding the correlation between O3 and GDM (Zhang et al., 2020), likely due to differences in study populations, exposure windows, and O3 concentrations.

The development of complex diseases like GDM is influenced not only by genetic and environmental factors but also by their interactions. In this study, we examined both genetic and environmental factors and assessed their interactions using an additive model. We found significant synergistic interactions between the rs2383208 AA genotype and O3, the rs10811661 TT genotype and O3, and the rs10811661 TT genotype and PM2.5, all of which increase the risk of GDM. These findings suggest that pregnant women carrying these susceptible genotypes should minimize exposure to high concentrations of O3 and PM2.5, as the combined effect of these factors is greater than the sum of their individual impacts, and was associated with higher odds of GDM.

This study represents a large-scale case-control investigation on GDM in China, with strengths such as a sizable sample size and broad geographic coverage. We analyzed the relationship between SNPs and GDM using dominant, recessive, and additive models, and identified several GDM-related haplotypes through haplotype analysis. In our interaction analysis, both genetic and environmental factors were included, providing clinically relevant insights through an additive interaction model. In studying gene-environment (G × E) interactions, careful variable selection is essential for reliable inference, as widely recognized in the methodological literature (Zhou et al., 2021). Following the marginal analysis framework for G × E interactions proposed by Zhou et al. (2021), we restricted our analysis to SNPs with prior biological evidence of association with GDM. For all prespecified candidates, we evaluated main genetic effects, haplotype associations, and G × E interactions. SNPs that showed significant interactions also exhibited significant main effects in our study population, lending support to the variable-selection rationale. In contrast to hypothesis-free, high-dimensional genome-wide G × E scans, our targeted design focuses on a small number of biologically plausible SNPs, which increases statistical power and complements genome-wide approaches. This strategy facilitates the translation of G × E findings into actionable public health prevention and control efforts.

However, this study has several limitations. First, we assessed only three SNPs, which may have failed to capture additional variation within the target gene or nearby loci relevant to GDM. Second, the haplotype analysis was based on a limited set of SNPs, which may weaken the robustness of the findings. Third, the additive interaction analysis method only permits the examination of two interacting factors at a time, potentially overlooking the effects of more complex interactions. Prior work in type 2 diabetes indicates that G × E interaction in disorders of abnormal glucose metabolism are often nonlinear—that is, their strength varies with the level of environmental exposure—and this likely extends to GDM (Wu and Cui, 2013). In our study, exposures were dichotomized and analyzed under an additive model, precluding assessment of nonlinear interactions and potentially leading to under- or overestimation of effect sizes. Fourth, we adjusted for only three confounders (age, BMI, and parity), and unmeasured factors—such as smoking status, socioeconomic characteristics, and urban versus rural residence—may have influenced the estimates. Future studies should expand variant coverage, construct denser haplotype blocks, adopt models that can evaluate multifactor interactions, and adjust for a broader set of covariates to strengthen inference and facilitate clinical translation.

In conclusion, *CDKN2A/2B* gene polymorphisms, haplotypes, exposure to O_3_ and PM_2.5_, and gene-environment interactions all contribute to an increased risk of GDM in Chinese women.

## Data Availability

The datasets used and/or analyzed during the current study are available from the corresponding author on reasonable request.
