# Supplementary material for: CDKN2A/2B gene variants and gene-environment interactions increase the risk of gestational diabetes mellitus in Chinese women
Source: Front Cell Dev Biol. 2026 Apr 22;14:1725802. doi: 10.3389/fcell.2026.1725802 (PMC13144239; doi:10.3389/fcell.2026.1725802)
Supplement: Supplementary file 1 [file Table1.docx]

Supplementary Table 1 Association of three candidate SNPs of *CDKN2A/2B* gene with T2DM and GDM

| Number | SNP | *P-value* | OR(95%CI) | Disease | Reference |
| --- | --- | --- | --- | --- | --- |
| 1 | rs2383208 | 0.003 | 1.242（1.077-1.432） | GDM | Wang et al |
| 2 | rs10811661 | 0.017 | 1.22（1.04-1.44） | T2DM | Binh et al |
| 3 | rs10811661 | 0.0014 | 0.53（0.36-0.79） | GDM | Tarnowski et al |
| 4 | rs10811661 | 0.004 | 1.20（1.06-1.37） | T2DM | Wu et al |
| 5 | rs10811661 | 0.035 | 1.46（1.03-2.08） | GDM | Kasuga et al |
| 6 | rs1063192 | 0.003 | 1.418（1.143-1.908） | GDM | Wang et al |

| Gene(G) | Environment(E) | Case group | Control group | OR | Significance |
| --- | --- | --- | --- | --- | --- |
| ＋ | ＋ | ａ | ｂ | OR_ge_=ah/bg | G、E Joint action effect |
| ＋ | - | c | d | OR_g_=ch/dg | G acting on the effects alone |
| - | ＋ | e | f | OR_e_=eh/fg | G acting on the effects alone |
| - | - | g | h | 1 | Common control |

Supplementary Table 2 The rationale for the additive interactions

Supplementary Table 3 Clinical clinical characteristics of GDM versus healthy controls

|  | **Case（n=703）** | **Control（n=863）** | **Z/X^2^** | ***P-*value** |
| --- | --- | --- | --- | --- |
| BMI | 22.42±4.11 | 21.23±3.05 | -6.428 | **<0.01** |
| height | 160.84±4.88 | 160.75±4.93 | -0.511 | 0.609 |
| age | 30.60±3.93 | 30.08±3.65 | -2.211 | **0.027** |
| gravidity | 1.84±1.02 | 1.76±1.01 | -2.066 | **0.039** |
| FPG | 5.03±0.63 | 4.60±0.35 | -17.348 | **<0.01** |
| 1h-PG | 9.42±1.73 | 7.28±1.39 | -20.332 | **<0.01** |
| 2h-PG | 8.10±1.56 | 6.39±1.05 | -19.228 | **<0.01** |

Supplementary Table 4 The data on the allele and genotype frequencies of the 3 SNPs in the GDM and control group

| Chr | SNP | Minor allele | Major allele | Minor allele frequency | Genotype distribution* | Ho | He | *Р* HWE |
| --- | --- | --- | --- | --- | --- | --- | --- | --- |
| the GDM group | | | | | | | | |
| 9 | rs1063192 | G | A | 0.207 | 39/212/452 | 0.302 | 0.328 | 0.034 |
| 9 | rs2383208 | G | A | 0.383 | 107/325/271 | 0.462 | 0.473 | 0.557 |
| 9 | rs10811661 | C | T | 0.397 | 116/326/261 | 0.464 | 0.479 | 0.406 |
| the control group | | | | | | | | |
| 9 | rs1063192 | G | A | 0.200 | 37/272/554 | 0.315 | 0.320 | 0.622 |
| 9 | rs2383208 | G | A | 0.437 | 178/398/287 | 0.461 | 0.492 | 0.066 |
| 9 | rs10811661 | C | T | 0.469 | 203/403/257 | 0.467 | 0.498 | 0.067 |

* minor allele homozygotes / heterozygotes / major allele homozygotes

Supplementary Table 5 shows the linkage disequilibrium calculation

| **SNP** | **r^2^** | **D’** |
| --- | --- | --- |
| **rs1063192 - rs2383208** | 0.004 | 0.51 |
| **rs1063192 - rs10811661** | 0.003 | 0.44 |
| **rs2383208 - rs10811661** | **0.79** | **0.93** |

Supplementary Table 6 Relationship of three SNPs in the gene and GDM（mutant type was used as reference）

|  |  | **Case（n=703）** | **Control（n=863）** | ***P-*value** | **OR(95%Cl)** |
| --- | --- | --- | --- | --- | --- |
| **rs1063192** |  |  |  |  |  |
| Genotypes | AA | 452 | 554 | Ref | Ref |
|  | GA | 212 | 272 | 0.681 | 0.955（0.768-1.188） |
|  | GG | 39 | 37 | 0.281 | 1.292（0.810-2.060） |
| Allele | A | 1116 | 1380 | Ref | Ref |
|  | G | 290 | 346 | 0.688 | 1.036（0.870-1.234） |
| **rs2383208** |  |  |  |  |  |
| Genotypes | GG | 107 | 178 | Ref | Ref |
|  | AG | 325 | 398 | **0.032** | **1.358（1.026-1.799）** |
|  | AA | 271 | 287 | **0.002** | **1.571（1.173-2.103）** |
| Allele | G | 539 | 754 | Ref | Ref |
|  | A | 867 | 972 | **0.002** | **1.248（1.081-1.440）** |
| **rs10811661** |  |  |  |  |  |
| Genotypes | CC | 116 | 203 | Ref | Ref |
|  | TC | 326 | 403 | **0.012** | **1.416（1.080-1.856）** |
|  | TT | 261 | 257 | **0.000075** | **1.777（1.335-2.365）** |
| Allele | C | 558 | 809 | Ref | Ref |
|  | T | 848 | 917 | **0.000055** | **1.341（1.162-1.546）** |

Supplementary Table 7 Relationship of three SNPs in the gene and GDM（wild type was used as reference）

|  |  | **Case（n=703）** | **Control（n=863）** | ***P-Value*** | **OR(95%Cl)** |
| --- | --- | --- | --- | --- | --- |
| **rs1063192** |  |  |  |  |  |
| Genotypes | GG | 39 | 37 | Ref | Ref |
|  | GA | 212 | 272 | 0.221 | 0.739（0.456-1.200） |
|  | AA | 452 | 554 | 0.281 | 0.774（0.485-1.234） |
| Allele | G | 290 | 346 | Ref | Ref |
|  | A | 1116 | 1380 | 0.688 | 0.965(0.810-1.149) |
| **rs2383208** |  |  |  |  |  |
| Genotypes | AA | 271 | 287 | Ref | Ref |
|  | AG | 325 | 398 | 0.198 | 0.865（0.693-1.079） |
|  | GG | 107 | 178 | **0.002** | **0.637（0.476-0.852）** |
| Allele | A | 867 | 972 | Ref | Ref |
|  | G | 539 | 754 | **0.002** | **0.801（0.694-0.925）** |
| **rs10811661** |  |  |  |  |  |
| Genotypes | TT | 261 | 257 | Ref | Ref |
|  | TC | 326 | 403 | **0.048** | **0.797（0.636-0.998）** |
|  | CC | 116 | 203 | **0.000075** | **0.563（0.423-0.749）** |
| Allele | T | 848 | 917 | Ref | Ref |
|  | C | 558 | 809 | **0.00055** | **0.746（0.647-0.860）** |

Supplementary Table 8 The association of the constituent factors of PM2.5 with GDM

| Environmental factor | *P-Value* | OR(95%CI) | *P-Value** | OR(95%CI)* |
| --- | --- | --- | --- | --- |
| BC | **1.95E-7** | **1.343（1.202-1.501）** | **1.0E-9** | **1.322（1.181-1.479）** |
| NH_4_^+^ | **5.4656E-7** | **1.094（1.056-1.133）** | **3.0E-6** | **1.088（1.050-1.127）** |
| NO_3_^-^ | **3.1068E-7** | **1.056（1.034-1.078）** | **2.0E-6** | **1.052（1.031-1.075）** |
| OM | **1.2622E-7** | **1.056（1.035-1.078）** | **9.376E-7** | **1.053（1.031-1.075）** |
| SO_4_^2-^ | **3.0E-6** | **1.086（1.049-1.124）** | **0.00001** | **1.082（1.045-1.120）** |

*P-values and OR values were adjusted for BMI, age, and pregnancy
